# Psychological Biomarkers and Fibrosis: An Innovative Approach to Non-alcoholic Fatty Liver Disease

**DOI:** 10.3389/fmed.2020.585425

**Published:** 2020-10-22

**Authors:** Jesús Funuyet-Salas, María Ángeles Pérez-San-Gregorio, Agustín Martín-Rodríguez, Manuel Romero-Gómez

**Affiliations:** ^1^Department of Personality, Assessment, and Psychological Treatment, Faculty of Psychology, University of Seville, Seville, Spain; ^2^Unit for Clinical Management (UCM) Digestive Diseases and Ciberehd, Virgen del Rocío University Hospital, Institute of Biomedicine of Seville, University of Seville, Seville, Spain

**Keywords:** NAFLD, fibrosis, quality of life, mental health, coping, perceived social support

## Abstract

**Background:** It is unknown how perceived social support and the progression of liver damage influence the psychosocial profile of patients with non-alcoholic fatty liver disease (NAFLD). In the present study, we therefore investigated which biomarkers influence the quality of life, mental health, and coping strategies of NAFLD patients.

**Methods:** Quality of life (SF-12 and CLDQ-NAFLD), mental health (HADS and BDI-II), and coping strategies (COPE-28) were evaluated by high or low perceived social support (MSPSS) and the presence of non-alcoholic steatohepatitis (NASH) and significant fibrosis in 492 biopsy-proven NAFLD patients. The results were compared with quality of life normality tables for the general Spanish population. We also determined whether liver histology and biopsychosocial variables predicted participants' quality of life.

**Results:** Interactive effects were found in vitality (*p* = 0.05), activity (*p* = 0.005), anxiety (*p* = 0.04), and denial (*p* = 0.04), with NASH patients showing a higher-risk biopsychosocial profile when they perceived less social support. Furthermore, patients with low perceived social support showed lower quality of life, worse mental health, and more maladaptive coping than those with high perceived social support, regardless of NASH presence. Patients with significant fibrosis showed lower quality of life compared to those without or the general Spanish population. Patients with significant fibrosis also reported worse mental health and more maladaptive coping. Lastly, significant fibrosis, female sex, greater anxiety and depressive symptoms, and worse physical and mental health-related quality of life were found to be independent determinants of worse disease-specific quality of life in these patients.

**Conclusions:** Low perceived social support, significant fibrosis, and female sex were independently associated with a higher-risk psychosocial profile in NAFLD. These findings support the role of psychological biomarkers based on quality of life, mental health, and coping strategies in the management of these patients and suggest the potential benefits of a psychological intervention.

## Introduction

Non-alcoholic fatty liver disease (NAFLD) causes a stronger negative impact on patients' quality of life (QoL) than do viral, alcoholic, autoimmune, or cholestatic liver diseases (1, 2), especially impairing physical functioning or the ability to perform daily activities (3–6). Mental health is also affected by an increase in anxiety and depressive symptoms (7). Similarly, although coping strategies have not been studied in NAFLD, maladaptive coping, such as denial of the disease, anger or getting upset after the diagnosis, disengagement, or giving up (8), is often found among chronic liver patients. The influence of perceived social support on these variables has not been approached. However, in chronic liver diseases, such as hepatitis B or C, satisfactory support implies improved patient progress and recovery (9, 10) and a decrease in the frequency and intensity of depressive symptoms (11).

The fibrosis stage is the main predictor of mortality associated with NAFLD (12), although the results are contradictory. Some studies have found worse QoL in patients with non-alcoholic steatohepatitis (NASH) and advanced fibrosis than those with NAFLD without advanced fibrosis, with cirrhotic patients complaining of the most decline in their QoL of all severity levels (2, 4, 13). However, Huber et al. (14) did not find any significant effect of fibrosis stage on QoL. In addition, the relationship between fibrosis and mental health in NAFLD patients is not clear either. Several studies have found an association between the presence of fibrosis and anxiety and depressive symptoms (7, 15, 16), while Kim et al. (17) found no relationship. Furthermore, female sex has been associated with a worse physical and mental QoL than does male sex (2, 18).

In view of the shortage of psychological studies in NAFLD, we decided to analyze the differences in QoL, mental health, and the coping strategies of patients with the absence or presence of NASH by perceived social support (high or low). We also studied the influence of liver disease severity levels on these variables using data from the general Spanish population to compare QoL. Finally, we determined whether certain histological and biopsychosocial variables predicted participants' QoL. We hypothesized that patients would have worse QoL, more anxiety and depressive symptoms, and more maladaptive coping when they have low perceived social support, NASH, or significant fibrosis. Furthermore, we hypothesized that the presence of determinants of liver damage (moderate or severe steatosis, lobular inflammation, hepatocellular ballooning, and significant fibrosis) and a higher-risk biopsychosocial profile (female sex, older age, presence of obesity, worse physical and mental health-related QoL, greater anxiety and depressive symptoms, maladaptive coping strategies, and low perceived social support) would be associated with a greater negative impact on the disease-specific QoL of NAFLD patients.

## Materials and Methods

### Participants

**Figure 1:**
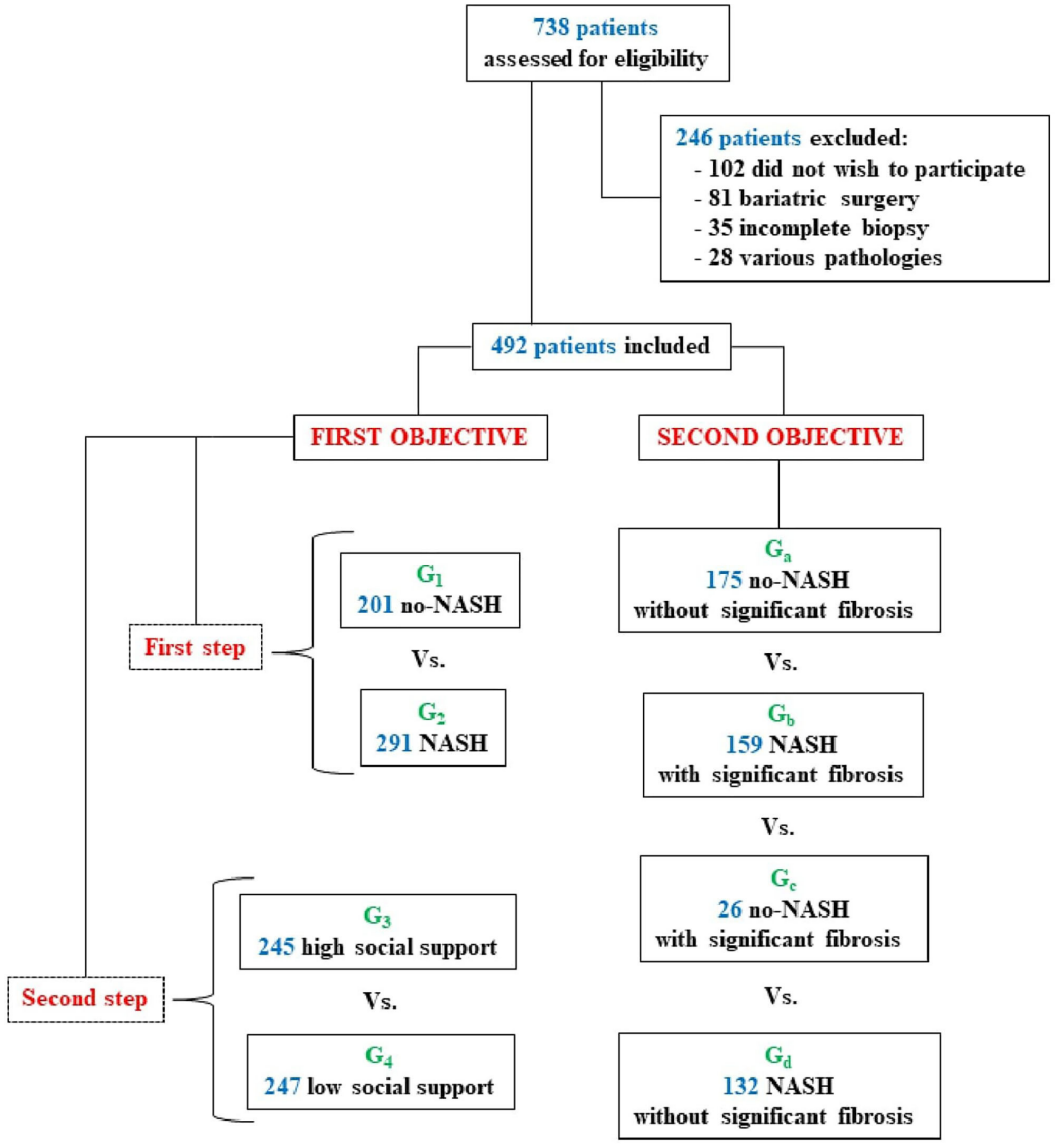
Study participant selection flowchart.

This research was approved by the Ethics Committee of the Virgen del Rocío University Hospital of Seville. All patients gave their informed consent for participation, and the research was conducted in accordance with the 1975 Declaration of Helsinki guidelines of good practice. As shown in Figure 1, we selected a group of 492 patients with biopsy-proven NAFLD (290 men and 202 women) with a mean age of 54.90 ± 11.74 years. The sociodemographic characteristics of the groups are shown in Supplementary Tables 1, 2. Data from the general Spanish population were also considered for QoL (SF-12) (19).

### Measures

#### The 12-Item Short-Form Health Survey (SF-12v.2)

This scale comprised 12 items with either three- or five-point Likert-type scales (20, 21). It evaluates the following eight dimensions of health-related QoL: physical functioning, role-physical, bodily pain, general health, vitality, social functioning, role-emotional, and mental health. It also calculates two summary measures using Quality Metric Health Outcomes^TM^ Scoring Software 5.0: physical component summary (PCS) and mental component summary (MCS). All scores ranged from 0 (worst state of health) to 100 (best state of health). Higher scores indicate better health-related QoL. In our sample, the Cronbach's alpha for the dimensions varied from 0.72 to 0.95. The Cronbach's alpha values for the PCS and MCS were 0.92 and 0.88, respectively (20).

#### Chronic Liver Disease Questionnaire—Non-alcoholic Fatty Liver Disease

The Chronic Liver Disease Questionnaire—Non-alcoholic Fatty Liver Disease (CLDQ-NAFLD) comprised 36 items rated on either a one- or a seven-point Likert-type scale (22). It evaluates specific QoL for NAFLD and NASH patients. It provides information referring to the total score on the scale and six domains: abdominal symptoms, activity, emotional, fatigue, systemic symptoms, and worry. Higher scores indicate better disease-specific QoL. In our sample, the Cronbach's alpha was 0.94 for the total instrument and from 0.68 to 0.89 for the domains.

#### Hospital Anxiety and Depression Scale

The Hospital Anxiety and Depression Scale (HADS) is made up of 14 items, seven on the anxiety subscale and seven on the depression subscale, with either zero- or three-point Likert-type scales (23). It evaluates anxiety and depressive symptoms. The test provides two total scores, one for anxiety and the other for depression. Scores range from 0 to 21 for each subscale. Higher scores indicate more anxiety and depressive symptoms. We used the Spanish version of this instrument (24). In our sample, the Cronbach's alpha was 0.81 for the anxiety subscale and 0.87 for the depression subscale.

#### Beck Depression Inventory—II

The Beck Depression Inventory—II (BDI-II) has 21 items answered on a four-point (0–3) scale, except for items 16 and 18, which have seven categories (25). It evaluates the severity of depression during the past 2 weeks. A total score of 0–63 is found. Higher scores show more severe depression. We used the Spanish version of this instrument (26). In our sample, the Cronbach's alpha was 0.91.

#### The Brief COPE

The Brief COPE (COPE-28) comprised 28 items with either zero- or three-point Likert-type scales (27). It evaluates 14 coping strategies: active coping, planning, instrumental support, emotional support, self-distraction, venting, disengagement, positive reframing, denial, acceptance, religion, substance use, humor, and self-blame. We used the Spanish version of this instrument (28). On all the subscales, higher scores indicate more use of the coping strategy. In our sample, the Cronbach's alpha was 0.80–0.99 on the different subscales, except for positive reframing which was 0.45.

#### Multidimensional Scale of Perceived Social Support

The Multidimensional Scale of Perceived Social Support (MSPSS) consists of 12 items with either a one- or a seven-point Likert-type scale (29). It evaluates perceived support from three different sources: family, friends, and partner or other significant persons. It also provides information on the total scale. We used the Spanish version of this instrument (30). Higher scores show more perceived social support. In our sample, the Cronbach's alpha was 0.94 for the total instrument and was 0.95–0.99 for the various dimensions of social support.

### Procedure

The 492 study patients were selected from 12 hospitals in six autonomous regions of Spain (Andalusia, Madrid, Castile and Leon, Catalonia, Cantabria, and Valencia). The same procedure was followed for all patients in the study. The researcher responsible at each participating hospital was contacted, and this contact provided a list of candidate NAFLD patients for the study. Then, these patients were phoned to make an appointment for the evaluation. All patients were evaluated by the same psychologist using the same psychological measures, always applied in the same order: a psychosocial interview and the SF-12, CLDQ-NAFLD, HADS, BDI-II, COPE-28, and MSPSS instruments. The inclusion criteria were as follows: (a) over 18 years of age; (b) informed consent; (c) no difficulties in understanding the evaluation instruments; (d) no severe or disabling psychopathological condition; and (e) diagnosed with biopsy-proven NAFLD. As all of the patients had undergone liver biopsy, they could be classified into groups by the hepatologist's criteria (G_1_ = absence of NASH and G_2_ = presence of NASH) and liver severity levels based on NASH and significant fibrosis (F2, F3, and F4) (G_a_ = no-NASH without significant fibrosis, G_b_ = NASH with significant fibrosis, G_c_ = no-NASH with significant fibrosis, and G_d_ = NASH without significant fibrosis) (Figure 1). Patients were classified as NASH or not after clinical assessment by the hepatologist, who provided a histological diagnosis by liver biopsy based on hepatocellular ballooning and lobular inflammation levels. The two perceived social support groups (G_3_ = high and G_4_ = low) were formed in two stages: firstly, each patient's total score on the MSPSS scale, which varied from 1 to 7, where higher scores show greater social support, was taken. These scores were then arranged in ascending order and the percentages accumulated were used to divide the sample into two groups at the 50.2 percentile (G_3_ = high and G_4_ = low) (Figure 1). Finally, to determine how liver histology and biopsychosocial variables predicted disease-specific QoL, the following histological variables provided by liver biopsy were analyzed: hepatic steatosis severity (a mild group of steatosis patients with 6–33% and a moderate-to-severe group with steatosis equal to or more than 34%); lobular inflammation (absence or presence depending on the number of foci per HPF, from none to more than one); hepatocellular ballooning (absence or presence based on the existence of ballooned cells); and significant fibrosis (absence, F0–F1, or presence, F2–F4, depending on the fibrosis stage). The biopsychosocial variables analyzed were sex (male or female), age, obesity, physical and mental health-related QoL (PCS and MCS; SF-12), anxiety and depressive symptoms (total anxiety and total depression; HADS, BDI-II), coping strategies (scores for all 14 coping strategies; COPE-28), and perceived social support (total scale score; MSPSS).

### Statistical Analysis

Pearson's chi-square test was applied to the sociodemographic variables to compare categorical variables (sex, marital status, education, and employment) and the *t*-test for independent samples or a one-way ANOVA (Welch's *U)* with Games–Howell *post hoc* pairwise analysis to compare the age variable.

A 2 × 2 factorial ANOVA (Snedecor's *F*) was performed to evaluate the influence on QoL (SF-12 and CLDQ-NAFLD), mental health (HADS and BDI-II), and coping strategies (COPE-28) exerted by NASH (absence or presence) and perceived social support (high or low). And to compare these variables (QoL, mental health, and coping strategies) between the NAFLD severity groups, a one-way ANOVA (Snedecor's *F* or Welch's *U*) as an omnibus test was computed depending on whether or not they met the assumption of homoscedasticity. For *post hoc* multiple comparisons, Tukey's honestly significant difference or the Games–Howell test was applied. The *t*-test for independent samples was also applied for comparison with the general Spanish population. Cohen's *d* (for continuous variables) and *w* (for categorical variables) were computed as effect size indexes.

Binary logistic regression analysis was used to determine the contribution of the histological and biopsychosocial variables to disease-specific QoL. The independent variables in the regression model were hepatic steatosis severity, lobular inflammation, hepatocellular ballooning, significant fibrosis, sex, age, obesity, PCS (SF-12), MCS (SF-12), total anxiety (HADS), total depression (HADS and BDI-II), the 14 coping strategies measured with the COPE-28, and total perceived social support (MSPSS). For categorical variables, reference groups were formed for patients with moderate to severe steatosis, presence of lobular inflammation, hepatocellular ballooning or significant fibrosis, female sex, and the presence of obesity. The total score on the CLDQ-NAFLD questionnaire (total CLDQ-NAFLD) was analyzed as the dependent variable. This score was arranged in ascending order and the cumulative percentages were used to divide the sample into two groups (better and worse QoL) at the 50th percentile. The results for binary logistic regression were reported as odds ratios (OR) at 95% confidence intervals. A two-sided *p-*value < 0.05 was considered statistically significant. All the data were analyzed with the SPSS Statistics v.25 program.

## Results

### Sociodemographic Variables

There were no important between-group differences (null or small effect sizes) in NASH, social support, or severity (Supplementary Tables 1, 2). The only difference in severity was that age was higher in G_b_ (NASH with significant fibrosis) than in G_d_ (NASH without significant fibrosis; *p* < 0.001, *d* = 0.557). Age was also higher in G_c_ (no-NASH with significant fibrosis) than in G_a_ (no-NASH without significant fibrosis; *p* = 0.003, *d* = −0.725) or G_d_ (NASH without significant fibrosis; *p* = 0.001, *d* = 0.778) (Supplementary Table 2).

### Influence of NASH and Social Support Variables on QoL, Mental Health, and Coping Strategies

**Figure 2:**
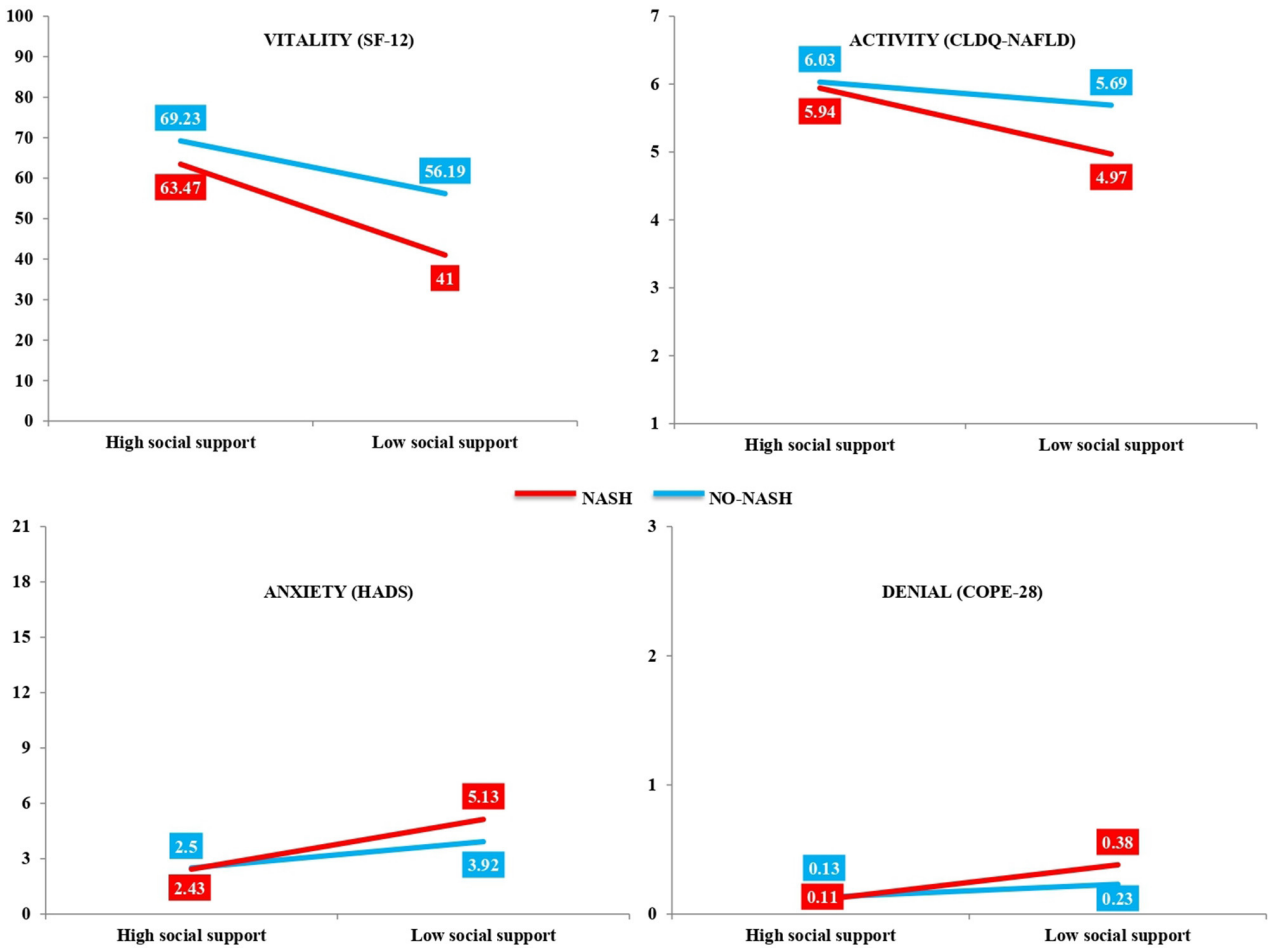
Interactive effects of non-alcoholic steatohepatitis (NASH; absence or presence) and level of social support (high or low) factors. Analysis of the influence of NASH and social support on the quality of life, mental health, and coping strategies of non-alcoholic fatty liver disease (NAFLD) patients showing interactive effects in vitality (*p* = 0.05), activity (*p* = 0.005), anxiety (*p* = 0.04), and denial (*p* = 0.04).

The results are shown in Table 1 (SF-12), Table 2 (CLDQ-NAFLD), Table 3 (HADS and BDI-II), and Table 4 (COPE-28). Four statistically significant interactive effects were found: vitality (*p* = 0.05; Table 1), activity (*p* = 0.005; Table 2), anxiety (*p* = 0.04; Table 3), and denial (*p* = 0.04; Table 4). As observed in Table 5 and Figure 2, the simple effects showed that NASH patients had less vitality (*p* < 0.001, *d* = 0.873), less activity (*p* < 0.001, *d* = 0.805), more anxiety (*p* < 0.001, *d* = −0.786), and more denial (*p* < 0.001, *d* = −0.638) when they perceived less social support. However, in patients without NASH, there were no differences depending on perceived social support, except in the vitality variable (*p* < 0.001, *d* = 0.505), which was lower in patients with low social support. Moreover, when social support was high, there were no differences between patients with and without NASH, but when social support was low, patients with NASH had lower scores in vitality (*p* < 0.001, *d* = 0.590) and activity (*p* < 0.001, *d* = 0.600).

**Table 1:** Quality of life (SF-12) of non-alcoholic fatty liver disease (NAFLD) patients based on non-alcoholic steatohepatitis (NASH; absence and presence) and social support level (high and low) variables.

**SF-12**	**NASH:** ***M***^a^ **(SD)**		**Social support level:** ***M***^a^ **(SD)**		**Main effects**		**Interaction effects**
	**Absence (G_**1**_)** ***n* = 201**	**Presence (G_**2**_)** ***n* = 291**	**High (G_**3**_):** ***n* = 245**	**Low (G_**4**_):** ***n* = 247**	**NASH:** ***F*_**(1,488)**_** ***p* (*d*^b^)**	**Social support level: *F*_**(1,488)**_** ***p* (*d*^b^)**	***F*_**(1,488)**_ (*p*)**
Physical functioning	76.85 (33.03)	65.71 (33.09)	81.73 (33.34)	60.83 (33.79)	13.53 <0.001 (0.337 S)	47.55 <0.001 (0.623 M)	3.73 (0.06)
Role-physical	81.53 (28.50)	73.21 (28.49)	86.67 (28.80)	68.08 (29.07)	10.17 0.002 (0.292 S)	50.76 <0.001 (0.642 M)	2.97 (0.09)
Bodily pain	79.18 (27.22)	70.86 (27.29)	81.43 (27.55)	68.61 (27.82)	11.09 0.001 (0.304 S)	26.33 <0.001 (0.463 S)	1.36 (0.24)
General health	52.06 (24.24)	46.72 (24.22)	56.77 (24.57)	42 (24.83)	5.74 0.02 (0.220 S)	43.97 <0.001 (0.598 M)	1.73 (0.19)
Vitality	62.71 (25.80)	52.24 (25.76)	66.35 (25.98)	48.59 (26.40)	19.62 <0.001 (0.406 S)	56.45 <0.001 (0.678 M)	3.98 (0.05)
Social functioning	88.85 (21.97)	85.57 (22.00)	94.43 (22.23)	79.99 (22.47)	2.63 0.11 (0.149 N)	51.01 <0.001 (0.646 M)	1.80 (0.18)
Role-emotional	83.02 (24.53)	78.74 (24.39)	89.31 (24.73)	72.45 (24.99)	3.65 0.06 (0.175 N)	56.45 <0.001 (0.678 M)	1.36 (0.24)
Mental health	73.00 (21.27)	68.01 (21.15)	77.60 (21.44)	63.41 (21.69)	6.57 0.01 (0.235 S)	53.07 <0.001 (0.658 M)	2.35 (0.13)
PCS	48.89 (10.21)	45.67 (10.06)	49.94 (10.17)	44.63 (10.37)	11.98 0.001 (0.318 S)	32.57 <0.001 (0.517 M)	2.51 (0.11)
MCS	52.26 (9.36)	50.57 (9.38)	54.61 (9.55)	48.21 (9.59)	3.86 0.05 (0.180 N)	55.44 <0.001 (0.669 M)	1.64 (0.20)

*A 2 × 2 factorial ANOVA (Snedecor's F) was applied*.

*SF-12, 12-Item Short-Form Health Survey; PCS, physical component summary; MCS, mental component summary*.

a *Higher scores show better quality of life*.

b *Effect sizes: N, null; S, small; M, medium*.

**Table 2:** Quality of life (CLDQ-NAFLD) of non-alcoholic fatty liver disease (NAFLD) patients based on non-alcoholic steatohepatitis (NASH; absence and presence) and social support level (high and low) variables.

**CLDQ-NAFLD**	**NASH:** *****M***^a^ (SD)**		**Social support level:** *****M***^a^ (SD)**		**Main effects**		**Interaction effects**
	**Absence (G_**1**_): *n* = 201**	**Presence (G_**2**_): *n* = 291**	**High (G_**3**_): *n* = 245**	**Low (G_**4**_): *n* = 247**	**NASH:** ***F*_**(1,488)**_** ***p* (*d*^b^)**	**Social support level: *F*_**(1,488)**_** ***p* (*d*^b^)**	***F*_**(1,488)**_ (*p*)**
Abdominal symptoms	5.64 (1.42)	5.51 (1.53)	5.88 (1.41)	5.28 (1.57)	0.91 0.34 (0.088 N)	19.36 <0.001 (0.402 S)	0.201 (0.65)
Activity	5.86 (1.28)	5.45 (1.19)	5.99 (1.25)	5.33 (1.26)	13.32 <0.001 (0.332 S)	33.92 <0.001 (0.526 M)	7.97 (0.005)
Emotional	5.90 (0.99)	5.62 (1.02)	6.17 (0.94)	5.35 (0.94)	9.14 0.003 (0.279 S)	82.23 <0.001 (0.872 L)	3.34 (0.07)
Fatigue	5.55 (1.13)	5.13 (1.19)	5.86 (1.25)	4.82 (1.26)	14.60 <0.001 (0.362 S)	89.58 <0.001 (0.829 L)	2.61 (0.11)
Systemic symptoms	5.98 (0.85)	5.64 (0.85)	6.10 (0.94)	5.52 (0.94)	17.60 <0.001 (0.400 S)	47.72 <0.001 (0.617 M)	1.04 (0.31)
Worry	6.30 (0.99)	6.10 (1.02)	6.39 (0.94)	6.00 (0.94)	5.22 0.02 (0.199 N)	19.42 <0.001 (0.415 S)	0.012 (0.91)
Total	5.87 (0.85)	5.58 (0.85)	6.06 (0.78)	5.38 (0.78)	14.54 <0.001 (0.341 S)	75.10 <0.001 (0.872 L)	3.04 (0.08)

*A 2 × 2 factorial ANOVA (Snedecor's F) was applied*.

a *Higher scores show better quality of life*.

b *Effect sizes: N, null; S, small; M, medium; L, large*.

**Table 3:** Mental health (HADS and BDI-II) of non-alcoholic fatty liver disease (NAFLD) patients based on non-alcoholic steatohepatitis (NASH; absence and presence) and social support level (high and low) variables.

	**NASH:** *****M***^a^ (SD)**		**Social support level: ***M***^a^ (SD)**		**Main effects**		**Interaction effects**
	**Absence (G_**1**_): *n* = 201**	**Presence (G_**2**_): *n* = 291**	**High (G_**3**_): *n* = 245**	**Low (G_**4**_): *n* = 247**	**NASH:** ***F*_**(1,488)**_** ***p* (*d*^b^)**	**Social support level: *F*_**(1,488)**_** ***p* (*d*^b^)**	***F*_**(1,488)**_ (*p*)**
**HADS**							
Total anxiety	3.20 (3.40)	3.78 (3.41)	2.46 (3.44)	4.52 (3.46)	3.29 0.07 (−0.170 N)	42.52 <0.001 (−0.597 M)	4.079 (0.04)
Total depression	2.26 (3.26)	3.09 (3.24)	1.22 (3.29)	4.13 (3.30)	7.66 0.006 (−0.255 S)	94.63 <0.001 (−0.883 L)	2.916 (0.09)
**BDI-II**							
Total depression	6.17 (6.95)	7.58 (6.99)	3.38 (7.04)	10.38 (7.07)	4.89 0.03 (−0.202 S)	12.69 <0.001 (−0.992 L)	2.272 (0.13)

*A 2 × 2 factorial ANOVA (Snedecor's F) was applied*.

a *Higher scores show worse mental health*.

b *Effect sizes: N, null; S, small; M, medium; L, large*.

**Table 4:** Coping strategies (COPE-28) of non-alcoholic fatty liver disease (NAFLD) patients based on non-alcoholic steatohepatitis (NASH; absence and presence) and social support level (high and low) variables.

**COPE-28**	**NASH:** *****M***^a^ (SD)**		**Social support level:** *****M***^a^ (SD)**		**Main effects**		**Interaction effects**
	**Absence (G_**1**_): *n* = 201**	**Presence (G_**2**_): *n* = 291**	**High (G_**3**_): *n* = 245**	**Low (G_**4**_): *n* = 247**	**NASH: *F*_**(1,488)**_*p* (*d*^b^)**	**Social support level: *F*_**(1,488)**_** ***p* (*d*^b^)**	***F*_**(1,488)**_ (*p*)**
Active coping	1.96 (0.71)	1.87 (0.68)	2.21 (0.78)	1.62 (0.78)	1.95 0.16 (0.129 N)	74.35 <0.001 (0.756 M)	0.10 (0.92)
Planning	1.29 (0.99)	1.34 (1.02)	1.63 (0.94)	1.01 (1.10)	0.27 0.60 (−0.050 N)	45.29 <0.001 (0.606 M)	1.35 (0.25)
Instrumental support	1.15 (0.99)	1.14 (1.02)	1.40 (0.94)	0.88 (0.94)	0.02 0.88 (0.010 N)	33.89 <0.001 (0.553 M)	0.33 (0.56)
Emotional support	1.03 (0.99)	1.10 (1.02)	1.33 (0.94)	0.79 (0.94)	0.56 0.45 (−0.070 N)	35.66 <0.001 (0.574 M)	0.10 (0.75)
Self-distraction	0.67 (0.99)	0.82 (1.02)	0.68 (0.94)	0.80 (0.94)	2.53 0.11 (−0.149 N)	1.83 0.18 (−0.128 N)	1.23 (0.27)
Venting	0.98 (0.99)	1.02 (1.02)	1.17 (1.09)	0.83 (1.10)	0.15 0.70 (−0.040 N)	13.55 <0.001 (0.310 S)	0.75 (0.39)
Disengagement	0.30 (0.57)	0.31 (0.51)	0.14 (0.47)	0.47 (0.47)	0.11 0.74 (−0.018 N)	50.74 <0.001 (−0.702 M)	0.19 (0.66)
Positive reframing	1.35 (0.99)	1.16 (1.02)	1.58 (0.94)	0.93 (0.94)	4.33 0.04 (0.189 N)	52.02 <0.001 (0.691 M)	0.47 (0.49)
Denial	0.18 (0.42)	0.24 (0.34)	0.12 (0.47)	0.30 (0.47)	2.63 0.10 (−0.157 N)	21.32 <0.001 (−0.383 S)	4.14 (0.04)
Acceptance	2.04 (0.71)	1.99 (0.68)	2.29 (0.78)	1.75 (0.78)	0.64 0.42 (0.072 N)	61.92 <0.001 (0.692 M)	1.44 (0.23)
Religion	0.84 (1.13)	0.99 (1.19)	0.90 (1.25)	0.94 (1.26)	2.00 0.16 (−0.129 N)	0.117 0.73 (−0.032 N)	0.01 (0.94)
Humor	1.11 (0.99)	1.00 (1.02)	1.29 (1.09)	0.82 (0.94)	1.45 0.23 (0.109 N)	24.21 <0.001 (0.462 S)	0.02 (0.89)
Self-blame	0.55 (0.71)	0.50 (0.68)	0.39 (0.78)	0.66 (0.78)	0.43 0.51 (0.072 N)	18.35 <0.001 (−0.346 S)	1.94 (0.16)

*A 2 × 2 factorial ANOVA (Snedecor's F) was applied*.

a *Higher scores show more use of the coping strategy*.

b *Effect sizes: N, null; S, small; M, medium*.

**Table 5:** Simple effects in vitality (SF-12), activity (CLDQ-NAFLD), anxiety (HADS), and denial (COPE-28).

		**Vitality (SF-12)**		**Activity (CLDQ-NAFLD)**		**Anxiety (HADS)**		**Denial (COPE-28)**	
		***p***	**Cohen's *d*^a^**	***p***	**Cohen's *d*^a^**	***p***	**Cohen's *d*^a^**	***p***	**Cohen's *d*^a^**
Social support level	High–low	Absence NASH (G_1_): *n* = 201							
		<0.001	0.505 M	0.05	0.283 S	0.004	−0.410 S	0.09	−0.250 S
		Presence NASH (G_2_): *n* = 291							
		<0.001	0.873 L	<0.001	0.805 L	<0.001	−0.786 M	<0.001	−0.638 M
NASH	Absence–presence	High social support (G_3_): *n* = 245							
		0.08	0.223 S	0.56	0.075 N	0.88	0.020 N	0.77	0.045 N
		Low social support (G_4_): *n* = 247							
		<0.001	0.590 M	<0.001	0.600 M	0.007	−0.352 S	0.01	−0.395 S

*SF-12, 12-Item Short-Form Health Survey; CLDQ-NAFLD, Chronic Liver Disease Questionnaire—Non-alcoholic Fatty Liver Disease; HADS, Hospital Anxiety and Depression Scale; COPE-28, Brief COPE*.

a *Cohen's d: N, null effect size; S, small effect size; M, medium effect size; L, large effect size*.

The main effects by relevant effect sizes (medium and large) were that, regardless of whether NASH was present or not, patients with low social support had worse QoL (SF-12 and CLDQ-NAFLD) than those with high social support on most of the variables, except bodily pain, abdominal symptoms, and worry, in which there were no differences between the two groups (Tables 1, 2). In mental health (HADS and BDI-II), patients with low social support had higher scores in anxiety (*p* < 0.001, *d* = −0.597) and depressive symptoms measured with both HADS (*p* < 0.001, *d* = −0.883) and BDI-II (*p* < 0.001, *d* = −0.992) (Table 3). And in coping strategies (COPE-28), by relevant effect sizes (medium), patients with low social support scored higher in disengagement (*p* < 0.001, *d* = −0.702) and lower in active coping (*p* < 0.001, *d* = 0.756), planning (*p* < 0.001, *d* = 0.606), instrumental support (*p* < 0.001, *d* = 0.553), emotional support (*p* < 0.001, *d* = 0.574), positive reframing (*p* < 0.001, *d* = 0.691), and acceptance (*p* < 0.001, *d* = 0.692) (Table 4).

### Influence of Liver Disease Severity on QoL, Mental Health, and Coping Strategies

**Figure 3:**
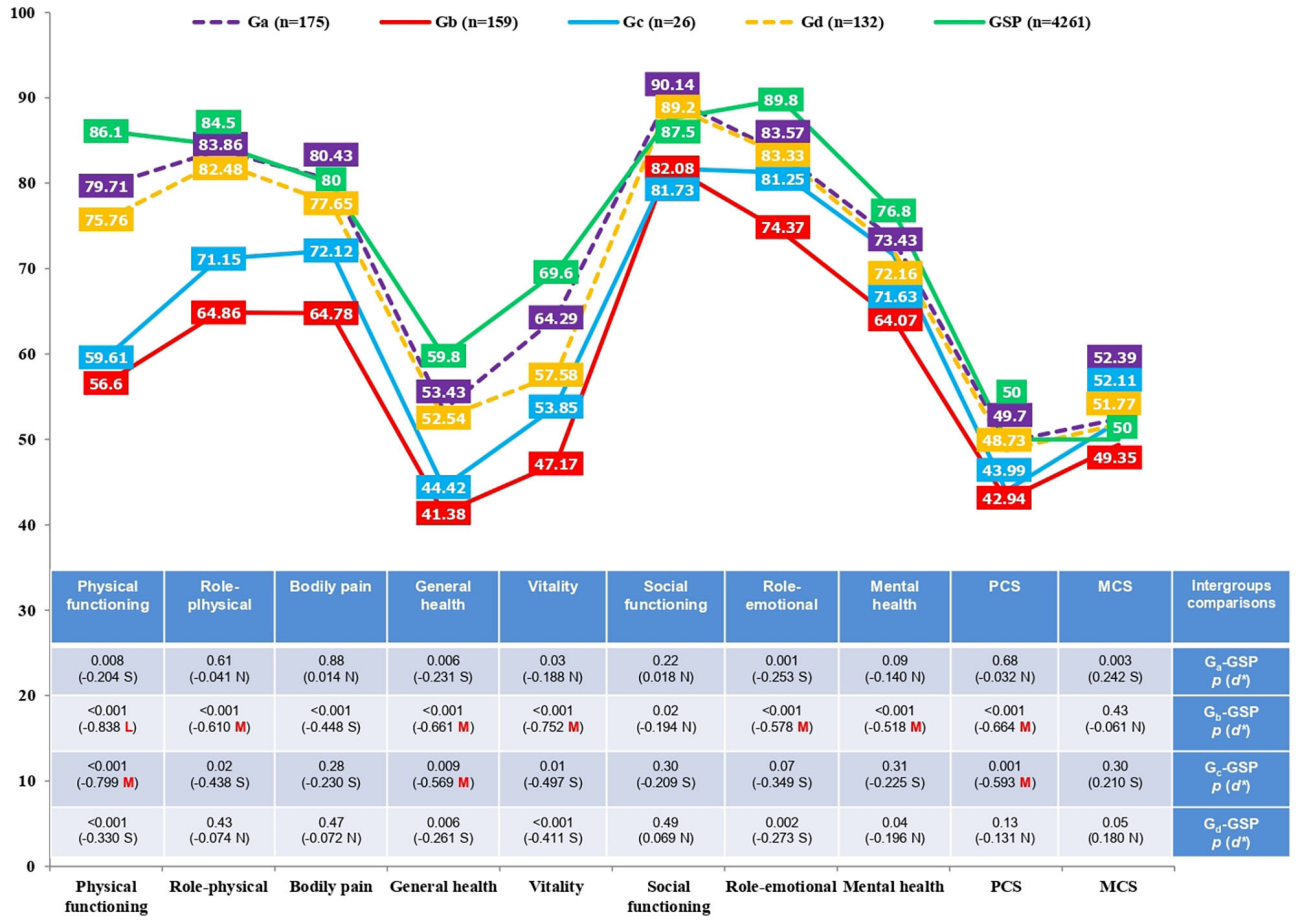
Comparison of quality of life (SF-12) in the non-alcoholic fatty liver disease (NAFLD) severity groups and the general Spanish population (GSP). *Cohen's *d*: *N*, null effect size; *S*, small effect size; *M*, medium effect size; *L*, large effect size. *G*_*a*_, no-NASH without significant fibrosis; *G*_*b*_, NASH with significant fibrosis; *G*_*c*_, no-NASH with significant fibrosis; *G*_*d*_, NASH without significant fibrosis; *GSP*, general Spanish population; *PCS*, physical component summary; *MCS*, mental component summary; *SF-12*, 12-Item Short-Form Health Survey.

**Figure 4:**
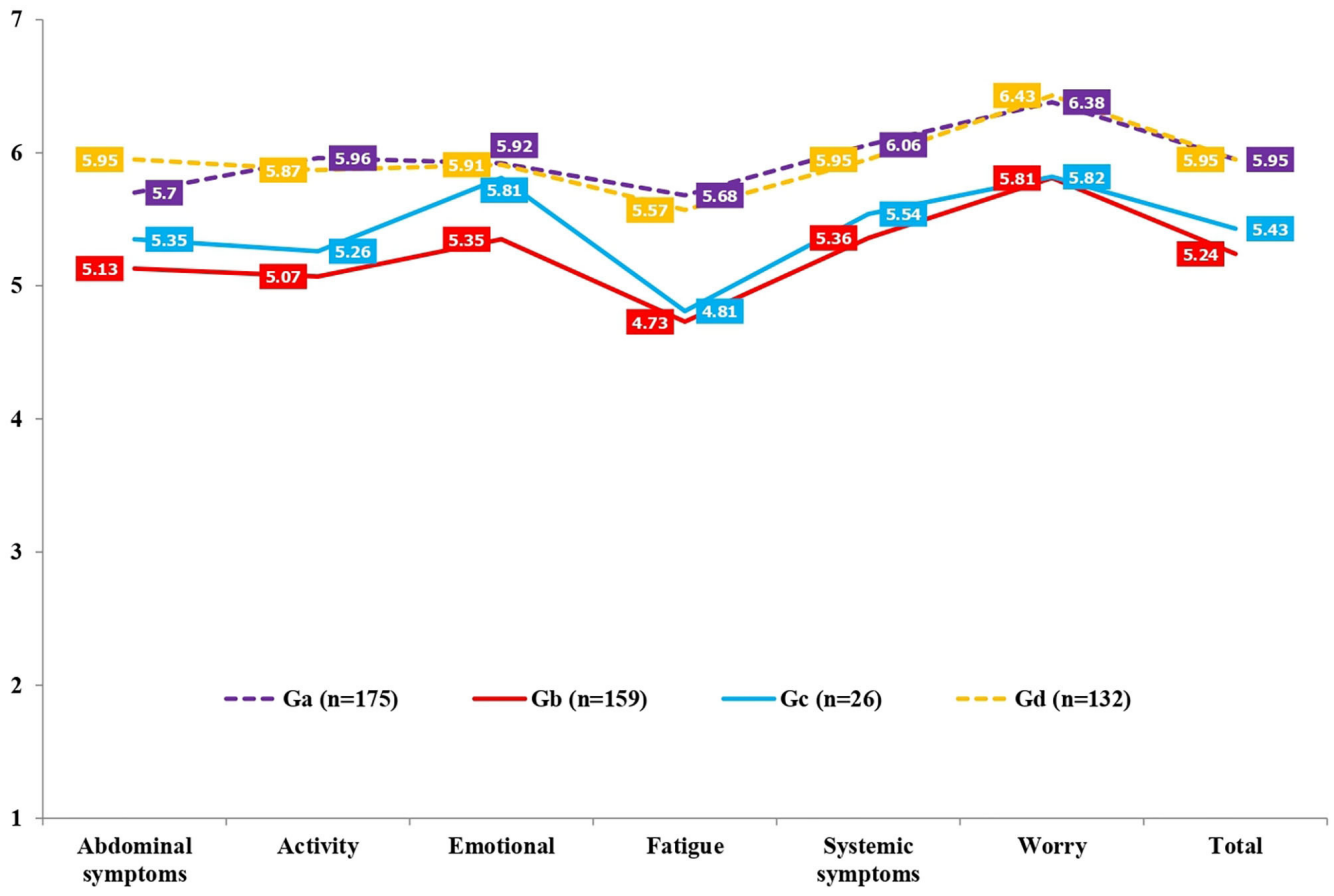
Comparison of quality of life (CLDQ-NAFLD) in non-alcoholic fatty liver disease (NAFLD) severity groups. *G*_*a*_, no-NASH without significant fibrosis; *G*_*b*_, NASH with significant fibrosis; *G*_*c*_, no-NASH with significant fibrosis; *G*_*d*_, NASH without significant fibrosis; *NASH*, non-alcoholic steatohepatitis; *CLDQ-NAFLD*, Chronic Liver Disease Questionnaire—Non-alcoholic Fatty Liver Disease.

In QoL (SF-12 and CLDQ-NAFLD), there were statistically significant differences between the severity levels in physical functioning (*p* < 0.001), role-physical (*p* < 0.001), bodily pain (*p* < 0.001), general health (*p* < 0.001), vitality (*p* < 0.001), social functioning (*p* = 0.01), role-emotional (*p* = 0.01), mental health (*p* = 0.001), PCS (*p* < 0.001), MCS (*p* = 0.03), abdominal symptoms (*p* < 0.001), activity (*p* < 0.001), emotional (*p* < 0.001), fatigue (*p* < 0.001), systemic symptoms (*p* < 0.001), worry (*p* < 0.001), and total CLDQ-NAFLD (*p* < 0.001). Specifically, by relevant effect sizes (medium and large), as shown in Tables 6, 7 and Figures 3, 4, G_a_ (no-NASH without significant fibrosis) scored higher in physical functioning (*p* < 0.001, *d* = 0.660), role-physical (*p* < 0.001, *d* = 0.603), bodily pain (*p* < 0.001, *d* = 0.566), vitality (*p* < 0.001, *d* = 0.638), PCS (*p* < 0.001, *d* = 0.647), activity (*p* < 0.001, *d* = 0.679), emotional (*p* < 0.001, *d* = 0.517), fatigue (*p* < 0.001, *d* = 0.736), systemic symptoms (*p* < 0.001, *d* = 0.750), worry (*p* < 0.001, *d* = 0.573), and total CLDQ-NAFLD (*p* < 0.001, *d* = 0.769) than G_b_ (NASH with significant fibrosis). G_a_ (no-NASH without significant fibrosis) also scored higher in physical functioning (*p* = 0.04, *d* = 0.610), PCS (*p* = 0.06, *d* = 0.574), activity (*p* = 0.06, *d* = 0.575), fatigue (*p* = 0.03, *d* = 0.671), systemic symptoms (*p* = 0.15, *d* = 0.514), worry (*p* = 0.14, *d* = 0.537), and total CLDQ-NAFLD (*p* = 0.11, *d* = 0.546) than G_c_ (no-NASH with significant fibrosis). G_b_ (NASH with significant fibrosis) scored lower in physical functioning (*p* < 0.001, *d* = −0.547), role-physical (*p* < 0.001, *d* =–0.579), PCS (*p* < 0.001, *d* = −0.542), abdominal symptoms (*p* < 0.001, *d* = −0.559), activity (*p* < 0.001, *d* = −0.633), emotional (*p* < 0.001, *d* = −0.521), fatigue (*p* < 0.001, *d* = −0.651), systemic symptoms (*p* < 0.001, *d* = −0.638), worry (*p* < 0.001, *d* = −0.633), and total CLDQ-NAFLD (*p* < 0.001, *d* = −0.804) than G_d_ (NASH without significant fibrosis). G_c_ (no-NASH with significant fibrosis) also scored lower in activity (*p* = 0.12, *d* = −0.523), fatigue (*p* = 0.06, *d* = −0.586), worry (*p* = 0.10, *d* = −0.594), and total CLDQ-NAFLD (*p* = 0.12, *d* = −0.570) than G_d_ (NASH without significant fibrosis). Similarly, the G_b_ (NASH with significant fibrosis) and G_c_ (no-NASH with significant fibrosis) groups differed considerably from the general Spanish population (GSP) in some dimensions of QoL measured with the SF-12 (Figure 3). More precisely, G_b_ (NASH with significant fibrosis) scored lower in physical functioning (*p* < 0.001, *d* = −0.838), role-physical (*p* < 0.001, *d* = −0.610), general health (*p* < 0.001, *d* = −0.661), vitality (*p* < 0.001, *d* = −0.752), role-emotional (*p* < 0.001, *d* = −0.578), mental health (*p* < 0.001, *d* = −0.518), and PCS (*p* < 0.001, *d* = −0.664) than the GSP. G_c_ (no-NASH with significant fibrosis) also scored lower in physical functioning (*p* < 0.001, *d* = −0.799), general health (*p* < 0.009, *d* = −0.569), and PCS (*p* < 0.001, *d* = −0.593) than the GSP.

**Table 6:** Comparison of quality of life (SF-12) in non-alcoholic fatty liver disease (NAFLD) severity groups: non-alcoholic steatohepatitis (NASH; with and without significant fibrosis) and no-NASH (with and without significant fibrosis).

**SF-12**	**G_a_–G_b_:** ***p* (*d*^a^)**	**G_a_–G_c_:** ***p* (*d*^a^)**	**G_a_–G_d_:** ***p* (*d*^a^)**	**G_b_–G_c_:** ***p* (*d*^a^)**	**G_b_–G_d_:** ***p* (*d*^a^)**	**G_c_–G_d_:** ***p* (*d*^a^)**
Physical functioning	<0.001 (0.660 M)	0.04 (0.610 M)	0.69 (0.127 N)	0.98 (−0.082 N)	<0.001 (−0.547 M)	0.14 (−0.489 S)
Role-physical	<0.001 (0.603 M)	0.26 (0.422 S)	0.99 (0.034 N)	0.79 (−0.189 N)	<0.001 (−0.579 M)	0.33 (−0.395 S)
Bodily pain	<0.001 (0.566 M)	0.57 (0.297 S)	0.79 (0.108 N)	0.68 (−0.238 S)	0.001 (−0.447 S)	0.83 (−0.190 N)
General health	<0.001 (0.480 S)	0.32 (0.373 S)	0.99 (0.036 N)	0.94 (−0.124 N)	0.001 (−0.439 S)	0.43 (−0.332 S)
Vitality	<0.001 (0.638 M)	0.26 (0.362 S)	0.14 (0.257 S)	0.65 (−0.220 S)	0.007 (−0.375 S)	0.92 (−0.125 N)
Social functioning	0.01 (0.335 S)	0.43 (0.143 N)	0.98 (0.012 N)	1.00 (0.013 N)	0.05 (−0.296 S)	0.54 (−0.314 S)
Role-emotional	0.01 (0.336 S)	0.97 (0.093 N)	1.00 (0.010 N)	0.58 (−0.255 S)	0.02 (−0.346 S)	0.98 (−0.088 N)
Mental health	0.001 (0.410 S)	0.98 (0.085 N)	0.96 (0.058 N)	0.38 (−0.220 S)	0.01 (−0.360 S)	1.00 (−0.026 N)
PCS	<0.001 (0.647 M)	0.06 (0.574 M)	0.81 (0.102 N)	0.97 (−0.094 N)	<0.001 (−0.542 M)	0.17 (−0.465 S)
MCS	0.03 (0.299 S)	1.00 (0.029 N)	0.95 (0.066 N)	0.55 (−0.267 S)	0.16 (−0.239 S)	1.00 (0.035 N)

*Tukey's honestly significant difference or Games–Howell was applied depending on whether or not they met the assumption of homoscedasticity*.

*G_a_, no-NASH without significant fibrosis; G_b_, NASH with significant fibrosis; G_c_, no-NASH with significant fibrosis; G_d_, NASH without significant fibrosis; PCS, physical component summary; MCS, mental component summary; SF-12, 12-Item Short-Form Health Survey*.

a *Effect sizes: N, null; S, small; M, medium*.

**Table 7:** Comparison of quality of life (CLDQ-NAFLD) between non-alcoholic fatty liver disease (NAFLD) severity groups: non-alcoholic steatohepatitis (NASH; with and without significant fibrosis) and no-NASH (with and without significant fibrosis).

**CLDQ-NAFLD**	**G_a_–G_b_:** ***p* (*d*^a^)**	**G_a_–G_c_:** ***p* (*d*^a^)**	**G_a_–G_d_:** ***p* (*d*^a^)**	**G_b_–G_c_:** ***p* (*d*^a^)**	**G_b_–G_d_:** ***p* (*d*^a^)**	**G_c_–G_d_:** ***p* (*d*^a^)**
Abdominal symptoms	0.006 (0.358 S)	0.73 (0.229 S)	0.37 (−0.186 N)	0.93 (−0.134 N)	<0.001 (−0.559 M)	0.30 (−0.412 S)
Activity	<0.001 (0.679 M)	0.06 (0.575 M)	0.91 (0.083 N)	0.91 (−0.138 N)	<0.001 (−0.633 M)	0.12 (−0.523 M)
Emotional	<0.001 (0.517 M)	0.94 (0.111 N)	1.00 (0.010 N)	0.15 (−0.428 S)	<0.001 (−0.521 M)	0.96 (−0.104 N)
Fatigue	<0.001 (0.736 M)	0.03 (0.671 M)	0.85 (0.095 N)	0.99 (−0.056 N)	<0.001 (−0.651 M)	0.06 (−0.586 M)
Systemic symptoms	<0.001 (0.750 M)	0.15 (0.514 M)	0.67 (0.131 N)	0.86 (−0.166 N)	<0.001 (−0.638 M)	0.33 (−0.409 S)
Worry	<0.001 (0.573 M)	0.14 (0.537 M)	0.94 (−0.066 N)	1.00 (−0.008 N)	<0.001 (−0.633 M)	0.10 (−0.594 M)
Total	<0.001 (0.769 M)	0.11 (0.546 M)	1.00 (0.000 N)	0.85 (−0.178 N)	<0.001 (−0.804 L)	0.12 (−0.570 M)

*Games–Howell post hoc pairwise analysis was applied*.

*G_a_, no-NASH without significant fibrosis; G_b_, NASH with significant fibrosis; G_c_, no-NASH with significant fibrosis; G_d_, NASH without significant fibrosis; CLDQ-NAFLD, Chronic Liver Disease Questionnaire—Non-alcoholic Fatty Liver Disease*.

a *Effect sizes: N, null; S, small; M, medium; L, large*.

As shown in Table 8, differences in mental health (HADS and BDI-II) were found in total anxiety (*p* = 0.01) and total depression in the HADS (*p* < 0.001) and BDI-II (*p* < 0.001). Specifically, by relevant effect sizes (medium), G_b_ (NASH with significant fibrosis) showed higher scores in total depression than groups G_a_ (no-NASH without significant fibrosis) (HADS: *p* < 0.001, *d* = −0.531; BDI-II: *p* < 0.001, *d* = −0.501) or G_d_ (NASH without significant fibrosis) (HADS: *p* < 0.001, *d* = 0.573; BDI-II: *p* < 0.001, *d* = 0.628).

**Table 8:** Comparison of mental health (HADS and BDI-II) between non-alcoholic fatty liver disease (NAFLD) severity groups: non-alcoholic steatohepatitis (NASH; with and without significant fibrosis) and no-NASH (with and without significant fibrosis).

		**No-NASH without significant fibrosis** **(G_a_): *n* = 175**	**NASH with significant fibrosis** **(G_b_): *n* = 159**	**No-NASH with significant fibrosis** **(G_c_): *n* = 26**	**NASH without significant fibrosis** **(G_d_): *n* = 132**	**Statistic**	***p***
		***M*^a^ (SD)**	***M*^a^ (SD)**	***M*^a^ (SD)**	***M*^a^ (SD)**		
HADS	Total anxiety	3.19 (3.37)	4.39 (3.95)	3.12 (3.94)	3.14 (3.41)	*U*_(3, 108.869)_ = 3.72	0.01
	Total depression	2.09 (2.94)	4.09 (4.44)	3.08 (4.49)	2.00 (2.62)	*U*_(3, 106.161)_ = 9.46	<0.001
BDI–II	Total depression	5.75 (7.11)	9.89 (9.28)	8.19 (8.26)	5.08 (5.57)	*U*_(3, 108.396)_ = 10.73	<0.001
		***Post hoc*** **comparisons**					
		**G**_a_**–G**_b_**:** ***p*** **(*****d***^b^**)**	**G**_a_**–G**_c_**:** ***p*** **(*****d***^b^**)**	**G**_a_**–G**_d_**:** ***p*** **(*****d***^b^**)**	**G**_b_**–G**_c_**:** ***p*** **(*****d***^b^**)**	**G**_b_**–G**_d_**:** ***p*** **(*****d***^b^**)**	**G**_c_**–G**_d_**:** ***p*** **(*****d***^b^**)**
HADS	Total anxiety	0.02 (−0.327 S)	1.00 (0.019 N)	1.00 (0.015 N)	0.43 (0.322 S)	0.02 (0.339 S)	1.00 (−0.005 N)
	Total depression	<0.001 (−0.531 M)	0.70 (−0.261 S)	0.99 (0.032 N)	1.71 (0.226 S)	<0.001 (0.573 M)	0.64 (0.284 S)
BDI-II	Total depression	<0.001 (−0.501 M)	0.49 (−0.317 S)	0.78 (0.105 N)	0.77 (0.193 N)	<0.001 (0.628 M)	0.27 (0.441 S)

*A one-way ANOVA (Welch's U) with Games–Howell post hoc pairwise analysis were applied*.

*HADS, Hospital Anxiety and Depression Scale; BDI-II, Beck Depression Inventory—II*.

a *Higher scores show worse mental health*.

b *Effect sizes: N, null; S, small; M, medium*.

In coping strategies (COPE-28), shown in Table 9, differences were found in active coping (*p* < 0.001), planning (*p* = 0.03), disengagement (*p* < 0.001), positive reframing (*p* = 0.001), denial (*p* = 0.004), acceptance (*p* < 0.001), and humor (*p* = 0.02). By relevant effect sizes (medium), group G_d_ (NASH without significant fibrosis) scored lower in disengagement than groups G_c_ (no-NASH with significant fibrosis; *p* = 0.21, *d* = 0.511) or G_b_ (NASH with significant fibrosis; *p* < 0.001, *d* = 0.589). G_d_ (NASH without significant fibrosis) also scored higher than G_b_ (NASH with significant fibrosis) in active coping (*p* < 0.001, *d* = −0.567) and acceptance (*p* < 0.001, *d* = −0.586) (Table 10).

**Table 9:** Comparison of coping strategies (COPE-28) between non-alcoholic fatty liver disease (NAFLD) severity groups: non-alcoholic steatohepatitis (NASH; with and without significant fibrosis) and no-NASH (with and without significant fibrosis).

**COPE-28**	**No-NASH without significant fibrosis** **(G_a_): *n* = 175**	**NASH with significant fibrosis** **(G_b_): *n* = 159**	**No-NASH with significant fibrosis** **(G_c_): *n* = 26**	**NASH without significant fibrosis** **(G_d_): *n* = 132**	**Statistic**	***p***
	***M*^a^ (SD)**	***M*^a^ (SD)**	***M*^a^ (SD)**	***M*^a^ (SD)**		
Active coping	1.99 (0.77)	1.66 (0.87)	1.88 (0.78)	2.10 (0.67)	*U*_(3,110.482)_ = 7.99	<0.001
Planning	1.27 (1.10)	1.17 (1.05)	1.50 (1.00)	1.52 (0.99)	*F*_(3,488)_ = 3.02	0.03
Instrumental support	1.20 (0.99)	1.03 (0.99)	0.88 (0.96)	1.25 (1.05)	*F*_(3,488)_ = 1.93	0.12
Emotional support	1.05 (1.01)	0.98 (0.99)	0.94 (0.93)	1.22 (1.04)	*F*_(3,488)_ = 1.53	0.21
Self-distraction	0.68 (0.98)	0.75 (1.02)	0.58 (0.66)	0.90 (0.97)	*U*_(3,120.760)_ = 1.88	0.14
Venting	1.01 (1.02)	1.06 (1.06)	0.86 (0.98)	0.96 (1.00)	*F*_(3,488)_ = 0.37	0.77
Disengagement	0.27 (0.51)	0.45 (0.63)	0.42 (0.66)	0.15 (0.35)	*U*_(3,106.617)_ = 9.24	<0.001
Positive reframing	1.38 (1.01)	0.97 (1.06)	1.17 (1.17)	1.36 (1.00)	*F*_(3,488)_ = 5.35	0.001
Denial	0.17 (0.37)	0.34 (0.54)	0.23 (0.51)	0.14 (0.36)	*U*_(3,107.139)_ = 4.74	0.004
Acceptance	2.07 (0.75)	1.77 (0.90)	1.90 (0.81)	2.23 (0.65)	*U*_(3,109.615)_ = 9.05	<0.001
Religion	0.82 (1.13)	1.01 (1.19)	0.96 (1.08)	0.98 (1.23)	*F*_(3,488)_ = 0.79	0.50
Substance use	0.00 (0.07)	0.00 (0.04)	0.00 (0.00)	0.00 (0.00)	*F*_(3,488)_ = 0.35	0.78
Humor	1.14 (1.10)	0.83 (1.01)	0.96 (1.02)	1.18 (1.03)	*F*_(3,488)_ = 3.42	0.02
Self-blame	0.54 (0.65)	0.63 (0.76)	0.54 (0.56)	0.36 (0.63)	*U*_(3,120.760)_ = 1.88	0.14

*A one-way ANOVA (Snedecor's F or Welch's U) was applied depending on whether or not they met the assumption of homoscedasticity*.

*COPE-28, Brief COPE*.

a *Higher scores show more use of the coping strategy*.

**Table 10:** *Post hoc* comparison of coping strategies (COPE-28) between non-alcoholic fatty liver disease (NAFLD) severity groups: non-alcoholic steatohepatitis (NASH; with and without significant fibrosis) and no-NASH (with and without significant fibrosis).

**COPE-28**	**G_a_–G_b_:** ***p* (*d*^a^)**	**G_a_–G_c_:** ***p* (*d*^a^)**	**G_a_–G_d_:** ***p* (*d*^a^)**	**G_b_–G_c_:** ***p* (*d*^a^)**	**G_b_–G_d_:** ***p* (*d*^a^)**	**G_c_–G_d_:** ***p* (*d*^a^)**
Active coping	0.002 (0.402 S)	0.92 (0.142 N)	0.54 (−0.152 N)	0.56 (−0.266 S)	<0.001 (−0.567 M)	0.56 (−0.303 S)
Planning	0.82 (0.093 N)	0.74 (−0.219 S)	0.17 (−0.239 S)	0.46 (−0.322 S)	0.03 (−0.343 S)	1.00 (−0.020 N)
Instrumental support	0.41 (0.172 N)	0.45 (0.328 S)	0.97 (−0.049 N)	0.91 (0.154 N)	0.24 (−0.216 S)	0.33 (−0.368 S)
Emotional support	0.91 (0.060 N)	0.95 (0.113 N)	0.49 (−0.166 N)	1.00 (0.042 N)	0.19 (−0.236 S)	0.58 (−0.284 S)
Self-distraction	0.93 (−0.070 N)	0.89 (0.120 N)	0.22 (−0.226 S)	0.68 (0.198 N)	0.58 (−0.151 N)	0.17 (−0.386 S)
Venting	0.97 (−0.048 N)	0.91 (0.150 N)	0.98 (0.049 N)	0.81 (0.196 N)	0.87 (0.097 N)	0.97 (−0.101 N)
Disengagement	0.02 (−0.314 S)	0.68 (−0.254 S)	0.08 (0.274 S)	1.00 (0.046 N)	<0.001 (0.589 M)	0.21 (0.511 M)
Positive reframing	0.002 (0.396 S)	0.76 (0.192 N)	1.00 (0.020 N)	0.79 (−0.179 N)	0.008 (−0.378 S)	0.83 (−0.175 N)
Denial	0.008 (−0.367 S)	0.94 (−0.135 N)	0.91 (0.082 N)	0.77 (0.209 S)	0.002 (0.436 S)	0.84 (0.204 S)
Acceptance	0.005 (0.362 S)	0.75 (0.218 S)	0.19 (−0.228 S)	0.86 (−0.152 N)	<0.001 (−0.586 M)	0.22 (−0.449 S)
Religion	0.49 (−0.164 N)	0.94 (−0.127 N)	0.65 (−0.135 N)	1.00 (0.044 N)	1.00 (0.025 N)	1.00 (−0.017 N)
Substance use	0.97 (0.000 N)	0.95 (0.000 N)	0.76 (0.000 N)	0.99 (0.000 N)	0.95 (0.000 N)	1.00 (0.000 N)
Humor	0.04 (0.294 S)	0.84 (0.170 N)	0.99 (−0.037 N)	0.94 (−0.128 N)	0.03 (−0.343 S)	0.77 (−0.215 S)
Self-blame	0.07 (−0.127 N)	1.00 (0.000 N)	0.09 (0.281 S)	0.88 (0.135 N)	0.005 (0.387 S)	0.48 (0.302 S)

*Tukey's honestly significant difference or Games–Howell for post hoc multiple comparisons was applied depending on whether or not they met the assumption of homoscedasticity*.

*G_a_, no-NASH without significant fibrosis; G_b_, NASH with significant fibrosis; G_c_, no-NASH with significant fibrosis; G_d_, NASH without significant fibrosis; COPE-28, Brief COPE*.

a *Effect sizes: N, null; S, small; M, medium*.

### Histological and Biopsychosocial Predictors of QoL in NAFLD Patients

A binary logistic regression was performed to evaluate the effect of the histological (steatosis, lobular inflammation, hepatocellular ballooning, and fibrosis) and biopsychosocial (sex, age, obesity, physical and mental health-related QoL, anxiety and depressive symptoms, coping strategies, and perceived social support) variables on the disease-specific QoL of NAFLD patients. The logistic regression model was statistically significant (χ^2^ = 367.256, *p* < 0.001). The model explained 70.1% (Nagelkerke's *R*^2^) of the variance in QoL, with an accuracy index of 0.852. Sensitivity was 86.6% and specificity was 83.7%, while the positive and negative predictive values were 0.841 and 0.861, respectively. Of all predictor variables, only fibrosis, sex, total depression (BDI-II), PCS (SF-12), total anxiety (HADS), and MCS (SF-12) were independently associated with total CLDQ-NAFLD. On the one hand, a significant inverse association was found between significant fibrosis (OR = 0.500, 95% CI = 0.253–0.987, *p* = 0.04), female sex (OR = 0.500, 95% CI = 0.254–0.981, *p* = 0.04), total depression (OR = 0.758, 95% CI = 0.661–0.869, *p* < 0.001), and total anxiety (OR = 0.858, 95% CI = 0.758–0.971, *p* = 0.01) and QoL (Table 11). On the other hand, a significant direct association was found between PCS (OR = 1.174, 95% CI = 1.123–1.227, *p* < 0.001) and MCS (OR = 1.073, 95% CI = 1.022–1.125, *p* = 0.004) and QoL (Table 11).

**Table 11:** Binary logistic regression analysis with total CLDQ-NAFLD as the dependent variable.

**Variables**	**Total CLDQ-NAFLD**						
	**Coefficient**	**SE**	**AUC** **(CI)**	***P***	**OR**	**95% CI**	
						**Lower**	**Upper**
Significant fibrosis	–0.693	0.347	0.616 (0.566–0.666)	0.04	0.500	0.253	0.987
Sex	−0.694	0.344	0.614 (0.564–0.664)	0.04	0.500	0.254	0.981
Total depression BDI-II	−0.277	0.070	0.887 (0.858–0.916)	<0.001	0.758	0.661	0.869
PCS	0.160	0.023	0.789 (0.747–0.831)	<0.001	1.174	1.123	1.227
Total anxiety HADS	−0.154	0.063	0.785 (0.745–0.825)	0.01	0.858	0.758	0.971
MCS	0.070	0.02	0.768 (0.724–0.811)	0.004	1.073	1.022	1.125

*SE, standard error; AUC, area under the ROC curve; OR, odds ratio; CI, confidence interval; PCS, physical component summary; MCS, mental component summary; CLDQ-NAFLD, Chronic Liver Disease Questionnaire—Non-alcoholic Fatty Liver Disease; BDI-II, Beck Depression Inventory—II; HADS, Hospital Anxiety and Depression Scale*.

## Discussion

This study analyzed the differences in QoL, mental health, and coping strategies in NAFLD patients based on factors such as social support and the severity of liver damage (NASH and fibrosis). We also analyzed whether histological and biopsychosocial variables could predict the QoL of these patients. There were no important sociodemographic differences between the groups compared, except in the age, which was higher in patients with significant fibrosis than in those without significant fibrosis. This finding coincides with other studies (31, 32) which had already noted the relationship between older age and the presence of significant or advanced fibrosis.

An interaction between NASH and social support was found in vitality, activity, anxiety, and denial. Among patients with NASH, those who reported low perceived social support showed less vitality and activity, greater anxiety, and more use of denial. This coincides with the results of a previous study done in patients with hepatitis C, which found a relationship between low levels of social support and more anxiety symptoms, as well as worse physical QoL (9). However, there were hardly any differences depending on social support in patients without NASH, except in vitality, which was higher in participants with high perceived social support.

When patients with low and high social support were compared, regardless of whether they had NASH or not, the first had poorer QoL and higher scores in anxiety and depressive symptoms, and more maladaptive coping, due to less use of strategies such as active coping, planning, using support, positive reframing, or acceptance. This ratifies the role of social support as a modulating agent of QoL, mental health, and coping strategies (33). Furthermore, low social support could be considered a major risk factor in NAFLD, especially when the disease progresses toward NASH and fibrosis. Therefore, it is fundamental to promote the creation of support networks like self-help groups because of their positive results in patient health, as already demonstrated in cancer (34), multiple sclerosis (35), or liver transplant candidate groups (36).

Moreover, patients with significant fibrosis had worse QoL in comparison with those without significant fibrosis and with the Spanish general population. This finding coincides with the study by David et al. (13), as it confirms the significant effect of fibrosis on QoL. In agreement with previous studies, the impact on QoL was mainly physical (3–6). Patients with significant fibrosis had particularly more impairment in physical functioning, role-physical, PCS, activity, emotional, fatigue, systemic symptoms, worry, and total CLDQ-NAFLD. This may be partially explained by the symptomatology associated with NAFLD, as problems affecting the patient's functionality, such as fatigue (3), daytime somnolence (37), and cognitive dysfunction (38), especially in the more advanced stages. These results agree with other studies comparing the QoL of NAFLD patients with that of the healthy population (3, 4, 13). We therefore suggest fibrosis as a determining factor in these differences, a conclusion confirmed by the results of the binary logistic regression analysis. Of all the variables analyzed for liver histology, fibrosis was the only one independently associated with QoL.

The relevance of sex in the QoL of NAFLD patients was also analyzed, with the results showing that female sex, along with the presence of significant fibrosis, was the main independent predictor of a worse QoL in these patients. Therefore, our study coincides with previous research, highlighting the greater vulnerability of the female sex to the impact caused by NAFLD (2, 18). Binary logistic regression analysis also revealed that the severity of anxiety and depressive symptoms predicted the QoL of the participants, in line with Huang et al. (39), who found that worse mental health was associated with a reduced QoL in chronic liver disease patients. Physical and mental health-related QoL, measured with the generic SF-12, also predicted disease-specific QoL measured with CLDQ-NAFLD, an instrument specific to NAFLD patients. As quality of life is one of the core goals of intervention in these patients, the model's predictive variables should be given special consideration in the future. Female patients with significant fibrosis, stronger anxiety and depressive symptoms, and worse physical and mental health-related QoL are more likely to have a greater impact on their health and well-being. These patients would therefore require closer attention in the design of multidisciplinary NAFLD management strategies. Lastly, fibrosis was also associated with worse mental health and more maladaptive coping strategies. NASH patients with significant fibrosis scored higher in depression than patients without significant fibrosis, whether or not they had NASH. Patients with significant fibrosis also employed maladaptive strategies, such as disengagement, to a greater extent in comparison with NASH patients without significant fibrosis, and fewer adaptive strategies such as active coping or acceptance. The results for mental health confirm the relationship between fibrosis and depression already noted previously by Weinstein et al. (15), Youssef et al. (7), and Tomeno et al. (16) and therefore contradict the conclusion of Kim et al. (17).

In brief, the main findings of this study verified that there are differences in the QoL, mental health, and coping strategies of NAFLD patients depending on the perceived social support and histological fibrosis and confirm that the relevant variables predicting a worse disease-specific QoL in these patients are significant fibrosis, female sex, greater anxiety and depressive symptoms, and worse physical and mental health-related QoL. These results are relevant because such patients need to follow certain interventions based on lifestyle changes including diet, physical activity, and exercise to promote NASH resolution and fibrosis regression when losing weight. However, the probability of successful adherence to these guidelines is certainly low (40). Indeed, in the study by Vilar-Gómez et al. (41), just 10% of patients lost 10% of body weight in spite of including behavioral meeting bimonthly. Keeping in mind the influence that variables such as QoL (42), mental health (43), coping strategies (44), or perceived social support (45) exert on therapeutic adherence, the biopsychosocial risk factors found in this study could be associated with a negative impact on adherence to intervention guidelines in NAFLD patients.

A structured psychological intervention could therefore improve therapeutic adherence and, as a consequence, the patient's clinical evolution (46), requiring special attention those patients with a low social support, significant fibrosis, or of the female sex due to their greater tendency to show a higher-risk biopsychosocial profile. We therefore recommend the inclusion of cognitive–behavioral treatment in NAFLD interventions (47) with techniques such as: psychoeducation focusing on NAFLD and how it progresses, as patients are generally unaware of their disease and the long-term consequences to their health (48); cognitive restructuring to intervene on unrealistic expectations related to weight loss, significantly linked to quitting therapy (49); problem-solving strategies to cope with obstacles to weight loss or maintenance (50); reinforcing alternative behaviors to eating without being hungry, for instance using relaxation or distraction techniques (50); using self-report questionnaires about weight, physical activity, and diet (51); setting commitments and realistic personal goals about weight loss or physical activity (49); and controlling stimuli, for example, keeping high-fat foods out of reach and placing those recommended in an accessible place at home (49).

Our study showed several limitations. Firstly, the possible collinearity of fibrosis and age. Secondly, the cross-sectional design of the current study did not allow us to analyze changes in histological features over time and their impact on the psychological profile. Thirdly, we did not analyze how self-efficacy, an important variable in chronic liver diseases, could influence the QoL and mental health of NAFLD patients (8). Lastly, analysis of the impact of other pathologies such as type 2 diabetes, arterial hypertension, hypercholesterolemia, hypertriglyceridemia, cardiovascular disease, thyroid disease, or obstructive sleep apnea syndrome on the biopsychosocial profile of NAFLD patients would be of interest for future research. Nevertheless, the large sample of consecutive patients from real clinical practice in Spain may be considered a major strength of this study.

## Data Availability Statement

The datasets presented in this article are not readily available because they may compromise the privacy of study participants and may not be shared publicly. The public availability of the data is restricted by the Ethics Committee of the Virgen del Rocío University Hospital of Seville (Spain). Data are available upon request to the authors. Requests to access the datasets should be directed to Jesús Funuyet-Salas, jfunuyet1@us.es.

## Ethics Statement

The studies involving human participants were reviewed and approved by Ethics Committee of the Virgen del Rocío University Hospital of Seville (Spain). The patients/participants provided their written informed consent to participate in this study.

## Author Contributions

JF-S, MP-S-G, AM-R, and MR-G designed and conducted research, collected, analyzed, interpreted the data, and wrote the paper. All authors provided critical revision and approval of the manuscript and contributed equally to this article.

## Conflict of Interest

The authors declare that the research was conducted in the absence of any commercial or financial relationships that could be construed as a potential conflict of interest.
