# Supplementary material for: Psychological Biomarkers and Fibrosis: An Innovative Approach to Non-alcoholic Fatty Liver Disease
Source: Front Med (Lausanne). 2020 Oct 22;7:585425. doi: 10.3389/fmed.2020.585425 (PMC7642831; doi:10.3389/fmed.2020.585425)
Supplement: Supplementary file 1 [file Data_Sheet_1.docx]

Supplementary Material

**Supplementary Table 1. Comparison of sociodemogaphic variables** **by NASH (absence and presence) and social support level (high and low).** *Effect sizes: N, null effect size; S, small effect size. The *t*-test for independent samples (age), and Pearson’s Chi-square (categorical variables) were applied.

|  | **NASH** | | | | **Intergroup comparisons** | | | **Effect sizes*** |
| --- | --- | --- | --- | --- | --- | --- | --- | --- |
|  | **Absence (G_1_)**  ***n*=201** | | **Presence (G_2_)**  ***n*=291** | |  | | |  |
|  | ***M (SD)*** | | ***M (SD)*** | | ***t* (*p*)** | | | **Cohen’s *d*** |
| Age | 54.05  (11.62) | | 55.49  (11.81) | | *t*_(1,490)_ = -1.332  (0.18) | | | -0.123 N |
|  | **%** | | **%** | | **χ^2^ (*p*)** | | | **Cohen’s *w*** |
| Sex   - Male - Female | 43.8  36.6 | | 56.2  63.4 | | *χ^2^*_(1)_=2.526  (0.11) | | | 0.072 N |
| Marital status   - With partner - Without partner | 41.6  38.3 | | 58.4  61.7 | | *χ^2^*_(1)_=0.364  (0.55) | | | -0.027 N |
| Education   - Low - Medium - High | 41.9  42  37.9 | | 58.1  58  62.1 | | *χ^2^*_(2)_=0.661  (0.72) | | | 0.037 N |
| Employment   - Employed - Unemployed | 45.2  36.8 | | 54.8  63.2 | | *χ^2^*_(1)_=3.614  (0.06) | | | 0.086 N |
|  | | **Social support level** | | | | **Intergroup comparisons** | **Effect sizes*** | |
|  | | **High (G_3_)**  ***n*=245** | | **Low (G_4_)**  ***n*=247** | |  |  | |
|  | | ***M (SD)*** | | ***M (SD)*** | | ***t* (*p*)** | **Cohen’s *d*** | |
| Age | | 53.29  (11.49) | | 56.50  (11.78) | | *t*_(1,490)_ = 3.052  (0.002) | -0.276 S | |
|  | | **%** | | **%** | | ***χ^2^* (*p*)** | **Cohen’s *w*** | |
| Sex   - Male - Female | | 52.1  46.5 | | 47.9  53.5 | | *χ^2^*_(1)_=1.459  (0.23) | -0.054 N | |
| Marital status   - With partner - Single | | 50.6  46.7 | | 49.4  53.3 | | *χ^2^*_(1)_=0.515  (0.47) | 0.032 N | |
| Education   - Low - Medium - High | | 41.5  50.3  62.9 | | 58.5  49.7  37.1 | | *χ^2^*_(2)_=15.065  (0.001) | 0.175 S | |
| Employment   - Employed - Unemployed | | 60.7  39.5 | | 39.3  60.5 | | *χ^2^*_(1)_=21.978  (<0.001) | -0.211 S | |

**Supplementary Table 2. Comparison of sociodemographic variables between NAFLD severity groups: NASH (with and without significant fibrosis) and no-NASH (with and without significant fibrosis).** *Effect sizes: N, null effect size; S, small effect size; M, medium effect size; L, large effect size. A one-way ANOVA (Welch´s *U*) with Games-Howell post-hoc pairwise analysis (age), and Pearson’s Chi-square (categorical variables) were applied.

|  | **No-NASH without significant fibrosis**  **(G_a_) *n*=175** | **NASH  with  significant  fibrosis**  **(G_b_) *n*=159** | **No-NASH with significant fibrosis**  **(G_c_) *n*=26** | **NASH  without  significant  fibrosis**  **(G_d_) *n*=132** | **Intergroup comparisons** | **Effect sizes*** | |
| --- | --- | --- | --- | --- | --- | --- | --- |
|  | ***M (SD)*** | ***M (SD)*** | ***M (SD)*** | ***M (SD)*** | ***U* _(3,114.284)_ (*p*)** | **Cohen’s *d*** | |
| Age | 53.07  (11.66) | 58.40  (9.99) | 60.65  (9.08) | 51.98  (12.87) | 12.93  (<0.001) | |  |
|  |  |  |  |  | G_a_-G_b_ (<0.001)  G_a_-G_c_ (0.003)  G_a_-G_d_ (0.87)  G_b_-G_c_ (0.66)  G_b_-G_d_ (<0.001)  G_c_-G_d_ (0.001) | | -0.491 S  -0.725 M  0.089 N  -0.236 S  0.557 M  0.778 M |
|  | **%** | **%** | **%** | **%** | ***χ^2^* (*p)*** | | **Cohen’s *w*** |
| Sex   - Male - Female | 38.3  31.7 | 28.3  38.1 | 5.5  5.0 | 27.9  25.2 | *χ^2^*_(3)_= 5.416  (0.14) | | 0.105 S |
| Marital status   - With partner - Single | 35.5  35.6 | 34.6  31.7 | 2.8  6.0 | 27.1  26.8 | *χ^2^*_(3)_= 1.813  (0.61) | | 0.061 N |
| Education   - Low - Medium - High | 34.6  39.2  33.3 | 36.9  30.8  26.5 | 7.4  2.8  4.5 | 21.2  27.3  35.6 | *χ^2^*_(6)_= 13.604  (0.03) | | 0.166 S |
| Employment   - Employed - Unemployed | 41.8  29.6 | 22.2  41.9 | 3.3  7.1 | 32.6  21.3 | *χ^2^*_(3)_= 29.073  (<0.001) | | 0.243 S |
